# From Bedside to Bot-Side: Artificial Intelligence in Emergency Appendicitis Management

**DOI:** 10.3390/life15091387

**Published:** 2025-09-01

**Authors:** Koray Ersahin, Sebastian Sanduleanu, Sithin Thulasi Seetha, Johannes Bremm, Cavid Abbasli, Chantal Zimmer, Tim Damer, Jonathan Kottlors, Lukas Goertz, Christiane Bruns, David Maintz, Nuran Abdullayev

**Affiliations:** 1 Department of General and Visceral Surgery, GFO Clinics Troisdorf, Academic Hospital of the Friedrich-Wilhelms-University Bonn, 50937 Troisdorf, Germany; 2 Department of Radiology and Neuroradiology, GFO Clinics Troisdorf, Academic Hospital of the Friedrich-Wilhelms-University Bonn, 53840 Troisdorf, Germany; 3 National Center for Oncological Hadrontherapy (CNAO), 27100 Pavia, Italy; 4 Institute for Diagnostic and Interventional Radiology, Faculty of Medicine and University Hospital Cologne, University of Cologne, 50937 Cologne, Germany; 5 Institute for Biomedical Engineering, Azerbaijan Technical University, AZ1073 Baku, Azerbaijan; 6 Faculty of Medicine, University of Cologne, 50391 Cologne, Germany; 7 Department of General, Visceral, Tumor and Transplantation Surgery, University Hospital of Cologne, Kerpener Straße 62, 50937 Cologne, Germany

**Keywords:** appendectomy, large language models, surgical decision making

## Abstract

**Introduction:** Acute appendicitis (AA) is a common cause of abdominal pain that can lead to complications like perforation and intra-abdominal abscesses, increasing morbidity and mortality, often requiring emergency surgery. Nevertheless, appendectomy is performed in up to 95% of uncomplicated cases, while complications like perforation and intra-abdominal abscesses increase morbidity and mortality. The current study compares the accuracy of GPT-4.5, DeepSeek R1, and machine learning in assisting with surgical decision-making for patients presenting with lower abdominal pain at the Emergency Department. **Methods:** In this multicenter retrospective study, 63 histopathologically confirmed appendicitis patients and 50 control patients with right abdominal pain presenting at the Emergency Department at two German hospitals between October 2022 and October 2023 were included. Using each patient’s clinical, laboratory, and radiological findings, DeepSeek (with and without Retrieval-Augmented Generation using 2020 Jerusalem guidelines) was compared in terms of accuracy with GPT-4.5 and a random forest-based machine-learning model, with a board-certified surgeon (reference standard) to determine the optimal treatment approach (laparoscopic exploration/appendectomy versus conservative antibiotic therapy). **Results:** Accuracy of agreement with board-certified surgeons in the decision-making of appendectomy versus conservative therapy increased non-significantly from 80.5% to 83.2% with DeepSeek and from 70.8 to 76.1% when GPT-4.5 was provided with the World Journal of Emergency Surgery 2020 Jerusalem guidelines on the diagnosis and treatment of acute appendicitis. The estimated machine-learning model training accuracy was 84.3%, while the validation accuracy for the model was 85.0%. **Discussion:** GPT-4.5 and DeepSeek R1, as well as the machine-learning model, demonstrate promise in aiding surgical decision-making for appendicitis, particularly in resource-constrained settings. Ongoing training and validation are required to optimize the performance of such models.

## 1. Introduction

Acute appendicitis (AA) is globally a common cause of lower abdominal pain with wide varying incidence according to sociodemographic region.

It often necessitates emergency department visits and emergency surgery, with up to 95% of patients with uncomplicated cases undergoing appendectomy [1].

According to long-term outcome update of 2021 on the Comparison of Outcomes of Antibiotic Drugs and Appendectomy (CODA) trial, antibiotic therapy is, in certain cases, a viable alternative to appendectomy for acute appendicitis, though nearly half of patients eventually require surgery within 4 years [2]. The choice between appendectomy and conservative (non-operative) therapy for acute appendicitis, still to this day, remains a topic of ongoing debate among surgeons.

Appendiceal perforation is a severe complication of AA, occurring in 16% to 40% of cases [3], with higher rates observed in very young and older patients. This complication significantly increases morbidity and mortality compared to non-perforated cases. In a specific cohort, perforation was noted in 13.8% of AA cases, predominantly affecting individuals aged 21–30 years, and was associated with high complication and mortality rates [4].

Another serious postoperative complication is intra-abdominal abscess, which occurs in 3% to 25% of cases following appendectomy, particularly after complicated appendicitis [5,6,7,8]. The risk factors for developing such abscesses remain controversial and appear to be similar between open and laparoscopic surgeries [9,10].

Diagnosing AA is complex and relies on a mix of clinical signs, patient age, vital signs, laboratory tests, and imaging techniques such as ultrasound or CT scans, depending on the clinical judgment and patient factors [11,12,13,14].

The decision between explorative laparoscopy/appendectomy and conservative treatment in cases of acute appendicitis (AA) should be made on an individual basis, with consideration of the patient’s unique clinical presentation. These decisions are now more frequently based on individual clinician assessments rather than standardized scoring systems, such as the Alvarado score [15], which has nevertheless been proven valuable in settings where diagnostic imaging is not immediately available. Consequently, there has been growing interest in the application of algorithms that utilize high-throughput, real-world data to aid in surgical decision-making.

Large language models (LLMs) are advanced artificial intelligence (AI) neural network architectures—often based on transformers—that are trained on vast amounts of textual data to generate human-like language and perform a variety of natural language processing tasks [16].

GPT, developed by OpenAI, exemplifies an LLM optimized for generalized language tasks and creative text synthesis [17], whereas DeepSeek [18] has been pre-trained on narrower, domain-specific datasets. In contrast to traditional machine-learning models [19] that generally rely on hand-engineered features for narrow, well-defined tasks, both GPT and DeepSeek demonstrate how deep learning can autonomously learn complex patterns from unstructured data, marking a significant evolution in the field of AI.

Recently introduced LLMs such as DeepSeek R1 and GPT-4.5 have shown enhanced reasoning capabilities and the potential in aiding clinicians by managing medical records, improving data interoperability, and supporting clinical decisions by providing summaries and translating medical records into standardized formats.

Previously, AI models focused on diagnosis and prognosis of acute appendicitis [20], while Sanduleanu et al. [21] explored agreement between GPT-3.5 and a machine-learning model with board-certified surgeons in surgical versus conservative management in suspected acute appendicitis.

Our current study additionally employs the recently released DeepSeek R1 and GPT-4.5 models with reasoning capabilities on this same patient population. It compares the accuracy differences of these models (GPT-4.5, DeepSeek R1, and machine learning) in assisting with surgical decision-making for patients presenting with lower abdominal pain at the Emergency Department.

## 2. Methods

This study received ethical approval (file number 23–1061-retro) from the Institutional Review Board (IRB) of GFO Kliniken Troisdorf on 10 February 2024, and informed consent was waived due to the retrospective nature of the study. No patient-identifying information was supplied to the artificial intelligence.

All examinations in this study involving human participants were conducted in accordance with the ethical standards of the national/institutional research committee and the Declaration of Helsinki from 1964.

### 2.1. Patient Characteristics and Study Criteria

Data from a total of *n* = 63 consecutive histopathologically confirmed appendicitis patients and *n* = 50 control patients presenting with right abdominal pain at the emergency department (ED) of two German hospitals (GFO Kliniken and UKK Cologne) were collected between October 2022 and October 2023.

For both groups, the following exclusion criteria were applied: (a) incomplete vital signs (temperature, blood pressure, and respiratory rate) at ED-admission; (b) missing physical examination findings; (c) missing inflammatory markers (CRP and leucocyte count); (d) missing findings from ultrasound examination for appendicitis cases that did not undergo an abdominal CT examination; (e) patient with contra-indication for abdominal surgery (e.g., intolerance general anesthesia).

Physical examination signs taken into account, as well as laboratory and radiological findings, have been described previously [21].

### 2.2. Study Design

Based on each patient’s clinical, laboratory, and radiological findings (full reports), DeepSeek (https://chat.deepseek.com) (accessed on 18 February 2025) and ChatGPT (https://chatgpt.com/) (accessed on 18 March 2025) were asked to determine the optimal course of treatment, namely laparoscopic exploration/appendectomy or conservative treatment with antibiotics using zero-shot prompting. Full .csv files with anonymized patient vital signs, physical examination, full text ultrasound, and computed tomography findings (where available) were provided to both DeepSeek and GPT-4.5. To enhance precision, LLM models can be tailored to identify the ideal treatment informed by up-to-date guidelines. This process, known as Retrieval-Augmented Generation (RAG), leverages external information (e.g., guidelines, keynote articles) to refine the model’s understanding. In the case of DeepSeek and GPT-4.5, the World Journal of Emergency Surgery 2020 Jerusalem guidelines [22] on the diagnosis and treatment of acute appendicitis were provided to the LLM.

The retrieval corpus consisted of the full-text PDF of the PubMed article.

DeepSeek and GPT-4.5 were prompted with the following question, upon providing it with the full .csv datasheet:

“Imagine you are an on-call surgeon on an Emergency Department of a Hospital and you are asked by your supervisor to provide a treatment recommendation for a list of patients with suspected appendicitis. Based on the information in the .CSV file alone, can you advise (generate a list for all 113 patients) per case if the patient should undergo laparoscopic exploration/appendectomy or whether conservative treatment with/without antibiotics is rather warranted?”

### 2.3. Statistical Analysis

Statistical analysis was performed using R, version 3.6.2, on RStudio, version 2023.03.0 + 386 (https://cran.r-project.org/) (accessed on 12 November, 2023). Overall agreement between DeepSeek R1 output and the reference standard was assessed by means of accuracy with the “Caret” and “irr” packages. To assess the level of agreement between LLM and the board-certified surgeons, Cohen’s kappa coefficient (κ) was used with the “psych” package. A kappa value of 0.61–0.80 was interpreted as substantial agreement, and values of 0.81–1.00 indicated almost perfect agreement.

We performed a power calculation (https://wnarifin.github.io/ssc/sskappa.html, accessed on 23 April 2025)) for Cohen’s kappa to detect a statistically significant agreement of κ = 0.8 (expected) versus a minimum acceptable κ = 0.4, assuming a 50% distribution between treatment categories, 5% significance level, 80% power, and no dropout, resulting in a required minimal sample size of 42 patients.

### 2.4. Machine-Learning Classifier

A random forest (RF) machine-learning classifier (default settings: 500 trees, mtry = √nr. of predictors, without internal cross-validation) was developed using variables such as age, vital signs, and diagnostic imaging (ultrasound and CT) to predict appendicitis in emergency department patients. The model was validated externally, and its predictive accuracy was assessed using an ROC curve, with McNemar’s Test comparing its performance against GPT-4.5 and DeepSeek according to a previously reported method in the study by Sanduleanu et al. [21]. The training cohort (*n* = 90) consisted of *n* = 50 appendicitis-confirmed cases and *n*= 40 controls from GFO Kliniken, while the validation cohort (*n* = 23) consisted of all *n*= 13 appendicitis-confirmed cases from UKK Cologne and *n* = 10 remaining controls from GFO Kliniken.

Statistical significance was defined as *p* < 0.05.

## 3. Results

**Figure 1:**
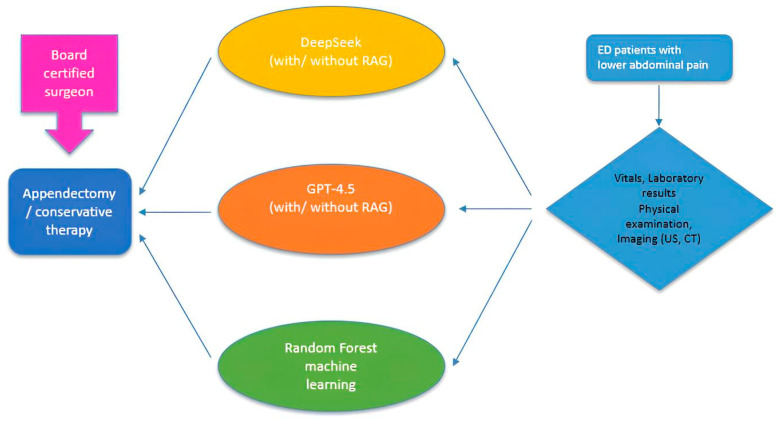
General study workflow.

The study encompassed 113 participants (63 histopathologically confirmed appendicitis cases and 50 control subjects) presenting with lower abdominal pain at the Emergency Department of GFO Kliniken and UKK Cologne. The analysis included mild to severely inflamed appendix cases. A study workflow is presented in Figure 1.

Within the GFO Kliniken cohort (*n* = 100, median age 35 years, 57% female), each patient underwent an ultrasound, and 29% received a CT scan. Clinical evaluations revealed an average of 1.12 appendicitis-associated signs (e.g., Psoas sign, Rovsign sign, McBurney/Lanz point tenderness) in affected patients, contrasting with an average of 0.24 signs in controls. The appendicitis group presented with an average temperature of 36.8 °C, CRP of 5.85 mg/dL, and leucocyte count of 12.82/μL; controls showed an average temperature of 36.6 °C, CRP of 1.19 mg/dL, and leucocyte count of 8.14/μL.

The UKK Cologne cohort (*n* = 13, median age 22 years, 38% female) demonstrated similar clinical findings with an average of 1.31 indicative signs. The average temperature was 36.5 °C, with CRP and leucocyte values recorded at 3.51 mg/dL and 13.43/μL, respectively.

Accuracy of agreement with board-certified surgeons in the decision-making of appendectomy versus conservative therapy increased from 80.5% to 83.2% with DeepSeek and from 70.8 to 76.1% when GPT-4.5 was provided with the World Journal of Emergency Surgery 2020 Jerusalem guidelines on the diagnosis and treatment of acute appendicitis [22].

The estimated machine-learning model training accuracy was 84.3%, while the validation accuracy for the model was 85.0%. This is in comparison to the DeepSeek accuracy of 83.2% with RAG, which did not perform significantly better than GPT-4.5 with RAG (McNemar *p* = 0.15) and was not significantly outperformed by the machine-learning model (McNemar *p* = 1.00).

The completed TRIPOD-AI standard checklist [23] can be found in the Supplementary Material.

**Figure 2:**
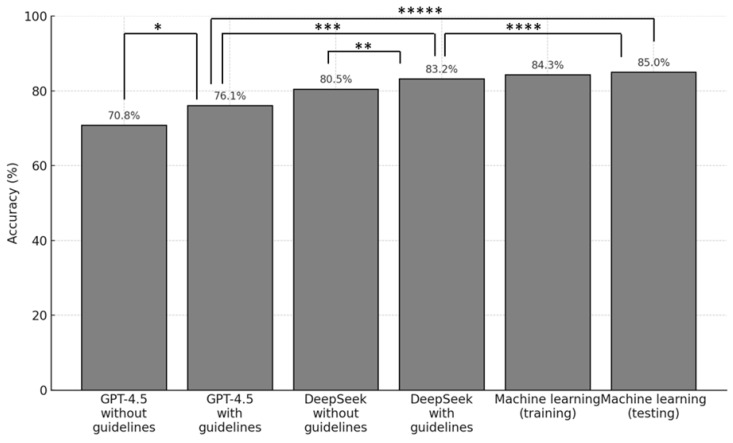
Accuracy comparison of GPT-4.5 versus DeepSeek versus machine-learning approach. * *p* = 0.15, ** *p* = 0.508, *** *p* = 0.15, **** *p* = 1.00, ***** *p* = 0.250.

A full summary of the accuracy metrics of the different models is presented in Figure 2.

Patient characteristics are presented in Table 1. While inter-observer Cohen Kappa (κ)-values are provided in Table 2.

## 4. Discussion

Although GPT-4.5 had lower overall accuracy than DeepSeek, it still showed reasonable concordance with board-certified surgeons, which highlights its potential as a decision-support tool for surgical decision-making in acute appendicitis. To the best of our knowledge, this study is the first in the literature to use the DeepSeek R1-based decision-support system for appendectomy.

Recent studies have explored the role of large language models in appendicitis-related clinical topics. Boyle et al. [23] evaluated ChatGPT-4, Google Gemini, Consensus, and Perplexity in supporting surgical guideline development for appendicitis, finding that while these models could assist with certain steps of guideline development (potentially reducing time and resource burden), they performed poorly in tasks requiring systematic literature searches and bias assessment. Ghanem et al. [24] assessed the quality and readability of AI-generated health information on appendicitis using ChatGPT-3.5, ChatGPT-4, Bard, and Claude-2, finding that while overall content quality was acceptable, sources were sometimes fabricated or missing entirely, and readability grade levels far exceeded recommended levels for the public.

Gracias et al. [25] evaluated ChatGPT’s performance in answering six appendicitis-related clinical questions formulated by RACS-qualified surgeons, comparing the model’s responses to current guidelines and expert surgeon assessments. They found that while ChatGPT could deliver timely and clinically relevant information, its performance was inconsistent and notably undermined by inaccuracies in generated references, underscoring the need for clinician oversight in using such tools.

OpenAI’s GPT-4.5 and DeepSeek R1 were selected in our current analysis because, at the time of study initiation, they represented two state-of-the-art large language models with advanced reasoning capabilities that were publicly accessible and supported multi-modal text-based data inputs. DeepSeek was chosen as a comparator to GPT-4.5 because of its open-source availability, cost efficiency, and reported competitive performance relative to proprietary LLMs, making it an attractive option for resource-limited healthcare settings.

The 2020 Jerusalem guidelines from the World Journal of Emergency Surgery were chosen as the reference guideline set due to their wide international acceptance, comprehensive recommendations for both diagnostic and therapeutic pathways in acute appendicitis, and clear, structured criteria. We considered them particularly relevant for this study as they include imaging-based decision-making and are frequently cited in contemporary surgical practice.

From our experiment, most noteworthy was that DeepSeek did not always accurately interpret the WSES guidelines, e.g., upon prompting, it did not recognize the flowchart and/or was not able to read the flowchart inside the WSES guideline that classifies patients into low, intermediate, and high risk according to Alvardo scores. This misalignment likely reflects the commonly rudimentary interpretation of figure-based workflows by current version LLMs, including GPT-4.5, which are primarily optimized for text-based reasoning and often cannot parse or apply the branching logic embedded in visual algorithms. Such limitations may be mitigated by converting flowchart content into structured, machine-readable text prior to prompting or integrating a medical-grade visual optical character recognition (OCR) tool into the workflow.

A notable observation in our cohort was that in unconfirmed cases on ultrasound and/or with low inflammation markers or negative findings upon physical examination, DeepSeek and GPT-4.5 often falsely recommend conservative treatment, while the surgeons, irrespective of the WSES guidelines (and perhaps based on high clinical suspicion) recommend more often explorative laparoscopy than what the guidelines may warrant. Another striking finding is that both DeepSeek and the machine-learning model outperform GPT-4.5 in terms of accuracy.

Several technical barriers were noted as well for real-time deployment of the LLMs in clinical settings. For instance, it was noted that upon prompting in the DeepSeek server, overload issues often ensued, leading to a “server busy” error that could not be solved by refreshing the page or logging in and out again. Secondly, LLM outputs can change with minor rephrasing of prompts, potentially producing inconsistent recommendations for the same patient data. Furthermore, sending identifiable patient data to external servers poses legal and ethical challenges under GDPR and HIPAA, unless models are deployed on secure, on-premise infrastructure.

DeepSeek is a Chinese AI startup company that has recently developed models like DeepSeek-V3 and DeepSeek-R1, which have been recognized for their cost efficiency and performance [26]. Native DeepSeek-R1 is trained using large-scale reinforcement learning without any supervised fine-tuning as a preliminary step [27]. This approach allows the model to develop reasoning capabilities through trial and error without relying on labeled data; such is the case with models from OpenAI.

Despite DeepSeek’s ability to analyze complex data inputs—from clinical presentation, physical examination, to ultrasound imaging results, and laboratory findings—it was still not able to generate thorough guideline-informed patient triage recommendations.

This study has several limitations. First, due to the retrospective nature of the study, not all physical examination and ultrasound reports were complete. Secondarily, shared decision-making by patient and doctor was not taken into account in the purely algorithmic decision-making process, nor the scheduling grid that may influence a surgeon’s decision, depending on factors such as available OR time and staff availability. Third, due to hallucinations, it is possible that DeepSeek and GPT-4.5 may give different answers if re-prompted, and it is unclear what exactly changes in the reasoning process of the algorithm after re-prompting. Fourth, the small number of cases in this study limits the generalizability and applicability of the findings to broader patient populations presenting with acute appendicitis.

The ability of DeepSeek and GPT-4.5 to perform surgical triage definitely poses ethical and legal concerns and can lead to a surgeon’s deprofessionalization. Under current legal and clinical governance frameworks, the ultimate accountability must remain with the treating surgeon, as LLMs cannot assume legal liability. For the surgeon to remain central in the decision process, LLMs should be deployed strictly as decision-support tools, offering guideline-informed recommendations while leaving the final decision to the surgeon’s clinical judgement. Safeguards could include mandatory human validation of all AI-generated outputs with clear institutional protocols defining the scope and limitations of AI assistance.

By providing consistent, guideline-informed recommendations [28], if thoughtfully implemented, tools like DeepSeek R1 and GPT-4.5 may support decision-making in the future in appendicitis management and the management of other acute abdominal conditions, reduce variability, and improve patient outcomes, particularly in settings with limited expertise or high case volumes. Surgical decision-making by LLMs holds the potential to also enhance patient education both before and after surgery by delivering accurate and relevant responses to FAQs about various surgical procedures [29].

Further research should also evaluate LLM data security and its implications for potential clinical applicability, clinician acceptance, potential biases related to stereotypes and gender aspects, potential fluctuations in training knowledge with LLM updates, prospective validation across multiple institutions, real-time trials with integrated AI triage tools, and potentially randomized comparisons with standard care and perceived patient satisfaction with LLM-aided treatment decisions.

## 5. Conclusions

GPT-4.5 and DeepSeek R1, as well as machine learning, demonstrate promise in aiding surgical decision-making for appendicitis, particularly in resource-constrained settings. Ongoing refinement and validation are required to optimize the performance of such models.

## Figures and Tables

**Table 1:** Patient characteristics per hospital cohort and training and testing set in the ML model. Abbreviations: ML—machine learning.

	GFO-Troisdorf Cohort (n = 100)	UKK Cologne Cohort (n = 13)	Training ML (n = 90)	Testing ML (n = 23)	Total (n = 113)
**Board-certified specialist decision**					
**Appendectomy (n)**	50	13	50	13	63
**Conservative (n)**	50	0	40	10	50
**Total (n)**	100	13	90	23	113
**Median age (years)**	35	22	35	23	34
**Gender**					
**Male (n)**	43	8	41	10	51
**Female (n)**	57	5	49	13	62
**Imaging upon ER-admission**					
**Ultrasound (%)**	100	100	100	100	100
**Computed Tomography (%)**	29	31	29	39	29

**Table 2:** Inter-observer Cohen Kappa (κ) between ML, DeepSeek and GPT-4.5.

Model Pair	Sample Size (n)	Cohen’s Kappa (κ)	Agreement Interpretation
**ML Testing vs. DeepSeek (with RAG)**	23	0.52	Moderate
**ML Testing vs. GPT-4.5 (with RAG)**	23	0.64	Substantial
**DeepSeek (with RAG) vs. GPT-4.5 (with RAG)**	113	0.75	Substantial

## Data Availability

The datasets analyzed in this current study are not publicly available due to patient privacy, but anonymized data will be available upon reasonable request to the corresponding author. Access to the data will be restricted to non-commercial research. The underlying code for this study (descriptive statistical analysis) is not publicly available but may be made available to qualified researchers upon reasonable request from the corresponding author. The data included in this manuscript have not been presented previously at any national or international meetings or conferences.

## Supplementary Materials

The following supporting information can be downloaded at: https://www.mdpi.com/article/10.3390/life15091387/s1. Reference [30] is cited in the Supplementary Materials.
